# Shaping electromagnetic waves using software-automatically-designed metasurfaces

**DOI:** 10.1038/s41598-017-03764-z

**Published:** 2017-06-15

**Authors:** Qian Zhang, Xiang Wan, Shuo Liu, Jia Yuan Yin, Lei Zhang, Tie Jun Cui

**Affiliations:** 1 State Key Laboratory of Millimeter Waves, Southeast University, Nanjing, 210096 China; 2 Synergetic Innovation Center of Wireless Communication Technology, Southeast University, Nanjing, 210096 China; 3 Cooperative Innovation Centre of Terahertz Science, No.4, Section 2, North Jianshe Road, Chengdu, 610054 China

## Abstract

We present a fully digital procedure of designing reflective coding metasurfaces to shape reflected electromagnetic waves. The design procedure is completely automatic, controlled by a personal computer. In details, the macro coding units of metasurface are automatically divided into several types (e.g. two types for 1-bit coding, four types for 2-bit coding, etc.), and each type of the macro coding units is formed by discretely random arrangement of micro coding units. By combining an optimization algorithm and commercial electromagnetic software, the digital patterns of the macro coding units are optimized to possess constant phase difference for the reflected waves. The apertures of the designed reflective metasurfaces are formed by arranging the macro coding units with certain coding sequence. To experimentally verify the performance, a coding metasurface is fabricated by automatically designing two digital 1-bit unit cells, which are arranged in array to constitute a periodic coding metasurface to generate the required four-beam radiations with specific directions. Two complicated functional metasurfaces with circularly- and elliptically-shaped radiation beams are realized by automatically designing 4-bit macro coding units, showing excellent performance of the automatic designs by software. The proposed method provides a smart tool to realize various functional devices and systems automatically.

## Introduction

In modern information science and technology, the printed microstrip reflectarray has been widely used as a kind of flat reflection-type antennas, which is generally composed of an array of radiating elements on a grounded substrate^1^. A lot efforts have been devoted to the researches of the printed microstrip reflectarray, which features the advantages of versatile radiation performances, compact structure, simple profile, and low cost as high-gain antennas^2^. Owning to such desirable features of traditional parabolic reflectors and planar phased arrays, it is more convenient for the reflectarray to produce the directive and shaped beams through the reflection phases of array elements^3^. In virtue of the diversity of element structures and relevant parameters, there are different reflection phases when the feeding source irradiates the microstrip patch elements^4–6^. Every element is independently controllable for the compensated phase of the phase delay caused by the path difference. However, one serious weakness of the printed microstrip reflectarray is the limited bandwidth^7^. Many techniques have been proposed to expand the working bandwidth, including using multilayer structure, true-time delay, subwavelength patch elements and dual-frequency phase synthesis method^8–12^.

On the other hand, various kinds of metasurfaces have been presented in recent years to control the radiation and scattered electromagnetic beams by designing gradient phases profiles on the metasurface, for example the anomalous reflection and refraction governed by the generalized Snell’s law^13–16^, polarization-state conversions^17–19^, conversions from spatial modes to surface modes^20^, optical-vortex generations^21–23^, and photonic spin hall effect^24–26^, etc. More recently, novel concepts of coding metasurface, digital metasurface, programmable metasurface, and reconfigurable reflectarray have been proposed^27–32^, which provide more freedoms and capabilities to control the electromagnetic waves and lights. However, most of the designs of reflectarrays and metasurfaces require many manual works (i.e. numerical simulations) to achieve the desired reflection phases of array elements.

To solve this problem, we propose in this work a software-based automatic-approach to provide an efficient way for automatic designs of unit cells using the optimization algorithm and commercial electromagnetic software. The particle swarm optimization (PSO) is an evolutionary computation model based on the swarm intelligence, which has been utilized in the function optimization, artificial neural network training, fuzzy control and other fields^33–36^. To solve the discrete combinatorial optimization problem, Kennedy and Eberhart have put forward the binary PSO (BPSO), whose particles are composed of binary encoding^37^. Because both discrete and continuous problems can be expressed by binary encoding, BPSO has much more practical application values^38, 39^. BPSO can be performed easily with profound intelligent background, and some improved algorithms have been developed in the scientific research and engineering applications^40–42^.

In this paper, we present software-automatically-designed unit cells based on the combination of discretely random square lattice, BPSO optimization, and electromagnetic computing software. We propose a software-based automatic-approach for the discretely random arrangement of micro coding units with the four-fold symmetry. The pattern of the micro coding units consists of many square metal sub-blocks, and different combinations of such sub-blocks will attach different phase compensations to the reflected waves. To get the desirable phase performance, we build up a bridge between the BPSO optimization algorithm and commercial software, CST Microwave Studio, to achieve the optimal arrangement of the square metal sub-blocks. On the basis of this method, we can obtain macro coding units of 1-bit coding elements with the phase difference of 180°, 2-bit coding elements with the phase difference of 90°, and so on, automatically over a broad operating frequency band. To verify the proposed method experimentally, we realize four-beam generations using the 1-bit coding elements, and circular/elliptical beam radiations using the automatically designed 4-bit coding elements with the constant phase difference of 22.5°. The numerical and experimental results are presented to confirm the good performance of the software-automatically-designed unit cells. The proposed method enables the automatic design of coding unit cells and provides us a smart tool for realizing multi-functional metasurface devices and systems.

## Results

### Micro macro coding units

**Figure 1:**
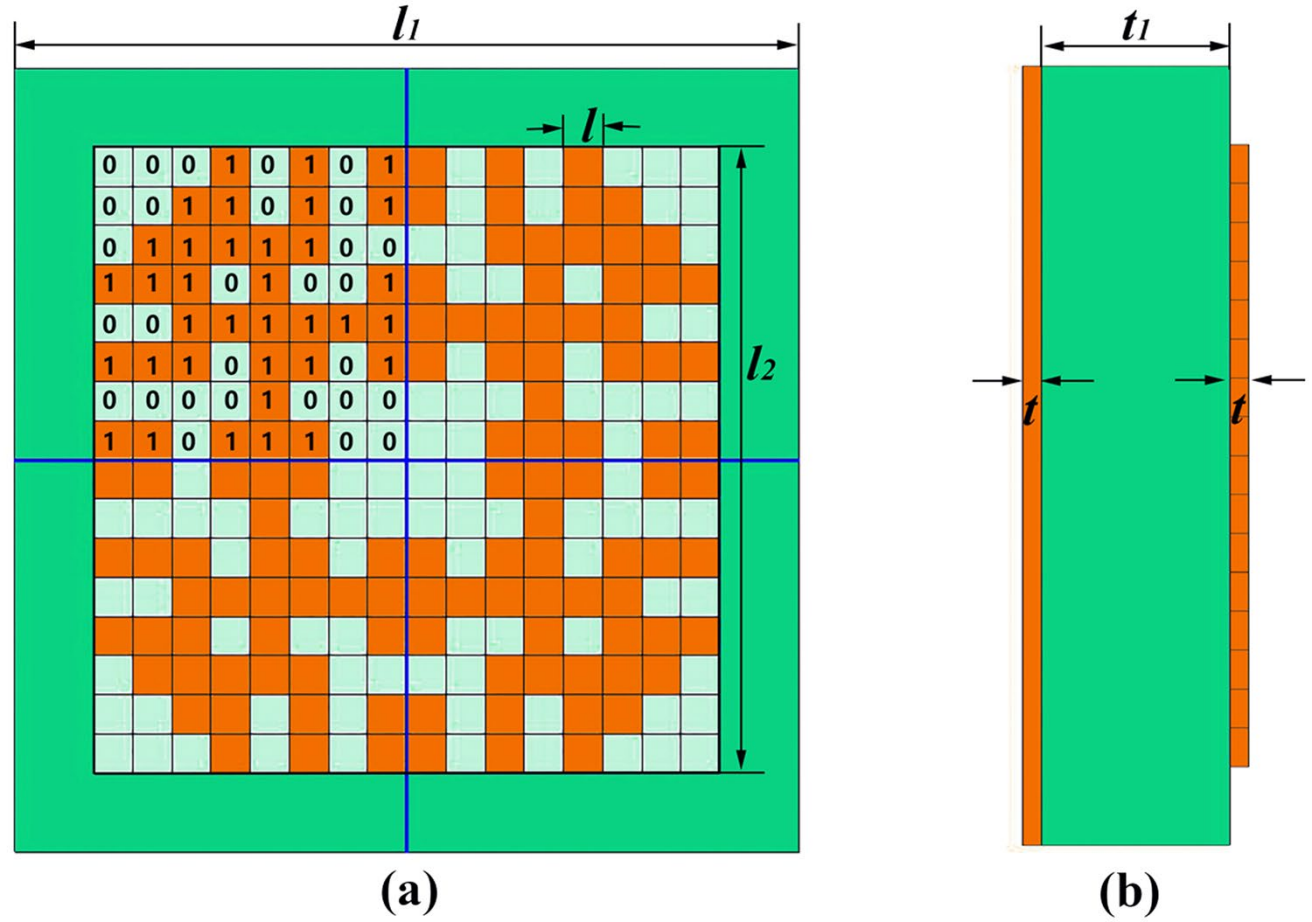
Geometry of the proposed micro coding unit cell, which is composed of 16 × 16 square sub-blocks. (**a**) The top view of the micro coding unit cell. (**b**) The cross-sectional view of the micro coding unit cell.

In order to realize the automatic designs of metasurface elements, we propose a software-based automatic-approach for the discretely random arrangement of micro coding units which is divided into 16 × 16 square sub-blocks with the four-fold symmetry imposed on the design, as illustrated in Fig. 1. Hence we just need to optimize one quarter of the unit cell in the automatic design. The 16 × 16 square sub-blocks are either covered with the metal thin film or not, in which the sub-blocks with metal are shown by yellow color, representing with ‘1’ in the BPSO particles, whereas the sub-blocks without metal are shown by white color, representing with ‘0’ in the BPSO particles, as demonstrated in Fig. 1(a). The parameters *l*_1_ = 10 mm and *l*_2_ = 8 mm are respectively chosen to describe the lengths of the square unit cell and the pattern, and hence the length of the sub-block is *l* = 0.5 mm. There is an obvious space between adjacent unit cells to decrease mutual coupling. The metallic patterns are printed on a commercial dielectric substrate, F4B, with the relative permittivity of 2.65 (1 + 0.003i) and thickness of *t*_1_ = 1 mm. The other side of the dielectric layer is the grounded substrate with annealed copper (electric conductivity *σ* = 5.8e + 007 S/m), and the thickness of the metallic layer is *t* = 0.018 mm, as depicted in Fig. 1(b).

Different compositions of ‘0’ and ‘1’ sub-blocks make it possible to realize the required amplitude and phase responses of unit cell. However, the manual sorting and designing of ‘0’ and ‘1’ sub-blocks is time-consuming, hard sledding, and has low efficiency owing to too many sub-blocks and too many arrangement possibilities. Here, we propose a software-based automatic-approach for the unit cell by optimizing the compositions of ‘0’ and ‘1’ sub-blocks using commercial software, CST Microwave Studio (MWS). To make automatic connection between the numerical simulations of unit cells and optimization algorithm, we firstly set up a bridge between the CST Microwave Studio and Matlab, the commercial mathematical software, in which CST MWS can call Matlab, and also be called by Matlab. When Matlab calls CST MWS, the CST MWS is the service system and Matlab is the management system. Because the two-dimensional array of ‘0’ and ‘1’ in Matlab has the capacity to tune the pattern of the unit cell, we can exploit MATLAB to create the ‘0’ and ‘1’ pattern and update the mode for CST MWS.

Here, the BPSO algorithm is utilized to achieve the optimal arrangement of the sub-blocks for our targets. In the PSO algorithm, the potential solution is regarded as a particle. In this BPSO algorithm, every particle is an *n*-dimensional vector, and every element of the vector is described by 0 or 1, for corresponding to a pattern of the unit cell. The speed of the particle indicates the probability of position change of the particle, which can be calculated analytically as

1 $${v}_{ij}^{k+1}=w{v}_{ij}^{k}+{c}_{1}\cdot ran{d}_{1}\cdot (pbes{t}_{ij}^{k}-{x}_{ij}^{k})+{c}_{2}\cdot ran{d}_{2}\cdot ({\rm{g}}bes{t}_{ij}^{k}-{x}_{ij}^{k})$$

where *rand*_1_ and *rand*_2_ are the random numbers between 0 and 1 in Matlab, *c*_1_ and *c*_2_ are the acceleration constants, *w* is the inertia coefficient, ${{\rm{pbset}}}_{ij}^{k}$ is the individual optimal position, and ${{\rm{gbset}}}_{ij}^{k}$ is the global optimal position. In this design, *x* donates whether the sub-block is metal or not. In virtue of the expression of probability, the value of the speed should be confined to the range of 0 and 1. The Sigmoid function is usually used for the mapping whose formula is

2 $$S({v}_{ij})=\frac{1}{1+\exp (-{v}_{ij})}$$

Therefore, the particles are assigned through

3 $${x}_{ij}=\{\begin{array}{ll}1 & rand < S({v}_{ij})\\ 0 & rand\ge S({v}_{ij})\end{array}$$

The targets to be optimized are then evaluated by the function *fitness*(*x*) looking for the best parameters.

**Figure 2:**
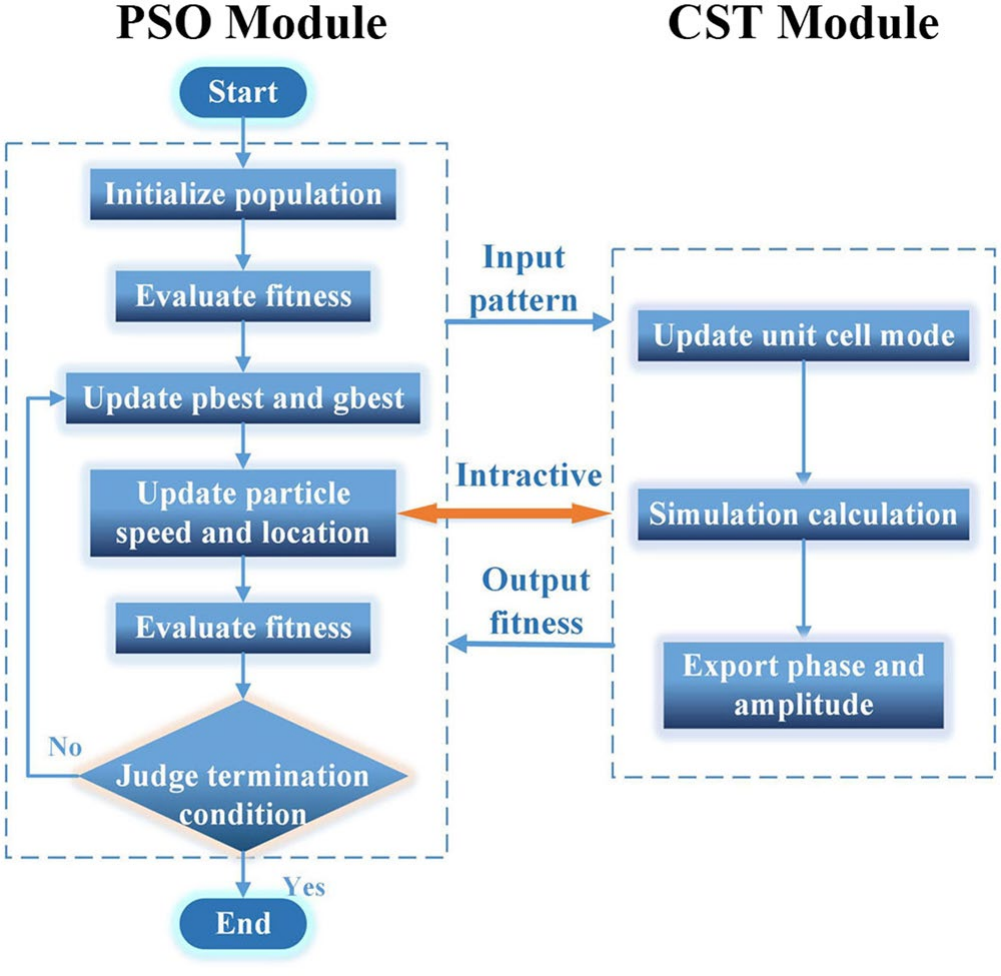
Flowchart of the BPSO algorithm together with the CST Microwave Studio. A bridge connects the CST Microwave Studio and Matlab, in which CST MWS can call Matlab, and also be called by Matlab.

Figure 2 depicts the automatic-design flow of the micro coding unit cell, in which we do no need any manual interventions, except setting the initial conditions and pressing the start button at the beginning. In the CST module, the unit cell mode without pattern should be built before running the algorithm. The initial population is a set of two-dimensional arrays just containing 0 and 1, which are randomly generated by Matlab. Inputting the pattern homologous array to the unit cell mode, we can acquire the phase and amplitude of every unit cell for evaluating the fitness to the required values. Then the PSO module updates the particle speed and the population location in each step of iterations, and sends the updated pattern to the execute simulation calculation by CST. At last, through multiple iterations and evaluating the fitness, it is convenient to get the optimal pattern automatically for the desired target.

**Figure 3:**
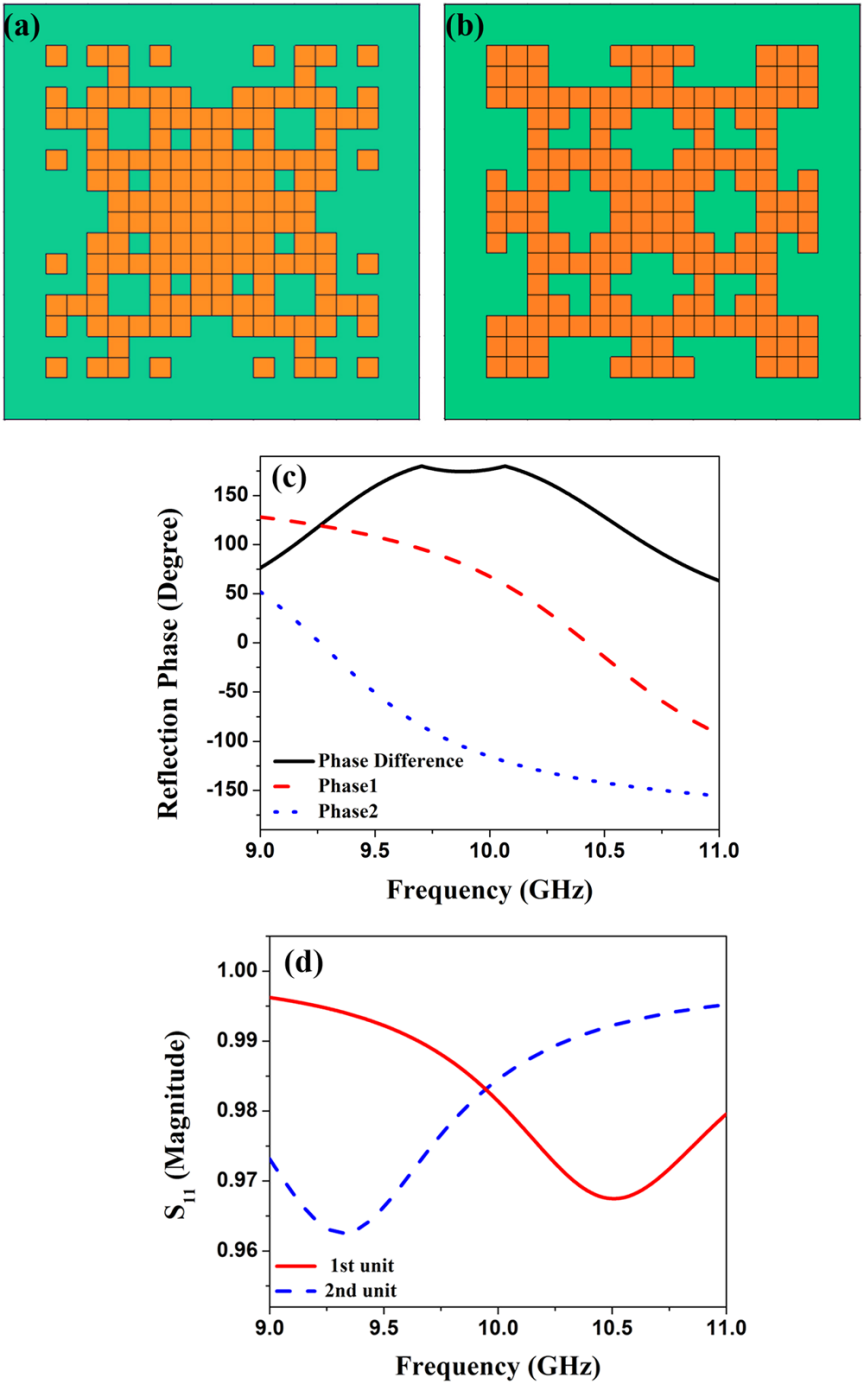
Models, reflection phases and amplitudes of the 1-bit coding unit cells. (**a**) The geometry of the first coding unit cell. (**b**) The geometry of the second coding unit cell. (**c**) The reflection phases of the 1-bit coding unit cells and their phase difference. (**d**) The reflection amplitudes of the 1-bit coding unit cells.

Because of the four-fold symmetry imposed on the design, the unit cell is not sensitive to the polarization direction for the normally incident waves. That is to say, there are the same responses for the transverse-electric (TE) and transverse-magnetic (TM) polarized waves, and hence we choose the TE polarized wave in all designs and simulations. As the first example, to obtain the broadband 1-bit micro coding units, the optimization target is set to the bandwidth for two unit cells with the phase difference of 180°. In order to simplify the optimization procedure, every particle is shown as a row vector containing 512 numbers of 0 or 1. The first half of the row vector is relative to the pattern of the first lattice unit cell, and the rest depicts the second lattice unit cell. The *fitness* function used for this optimization is

4 $$fitness={B}_{{180}^{\circ }\pm {10}^{\circ }}$$

representing the bandwidth of the phase difference between 170° and 190° within the X band. An initial population of 30 particles is evolved for a maximum of 100 iterations. As geometrically illustrated in Figs. 3(a) and (b), we finally get the two optimized 1-bit micro coding units. From the reflection phases and amplitudes shown in Figs. 3(c) and (d), it is expedient to observe that the phase difference of the two unit cells satisfies the criterion aforementioned in the frequency band from 9.5 GHz to 10.3 GHz, while their reflection amplitudes are very close to 1.

**Figure 4:**
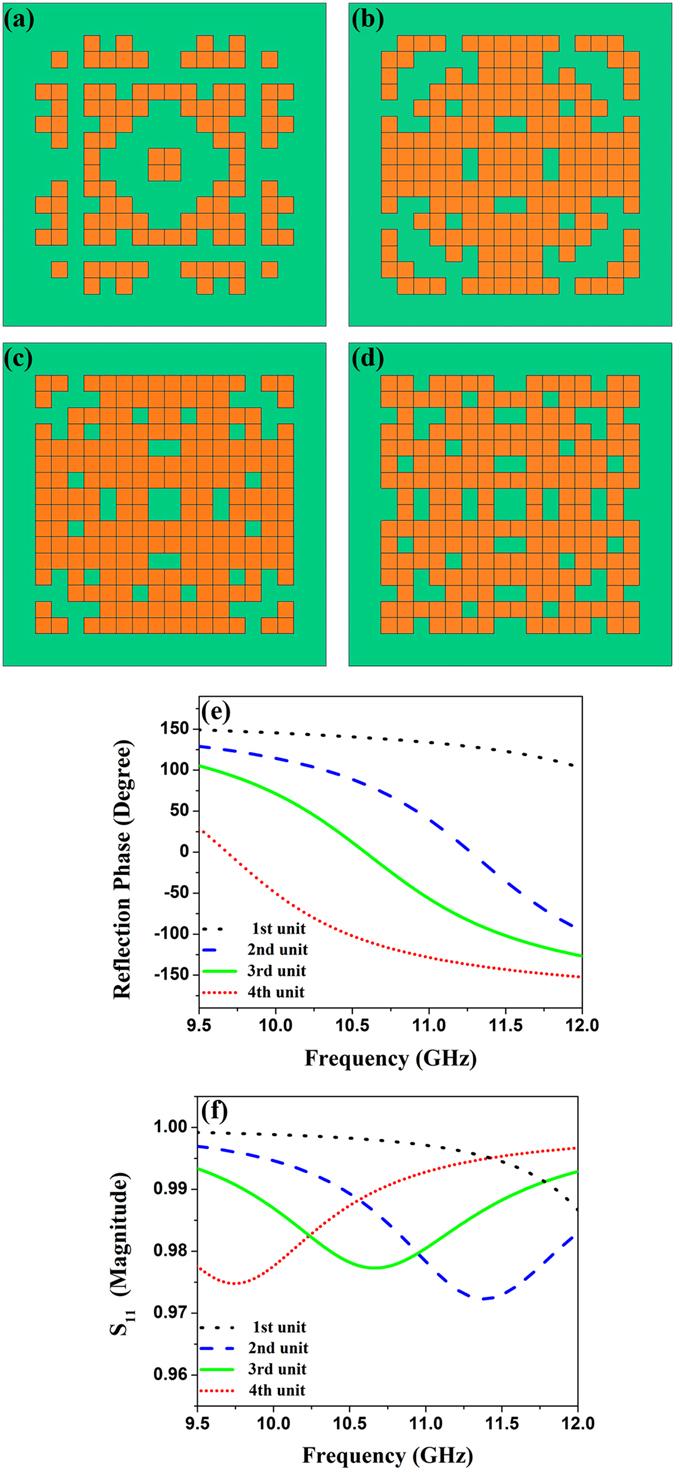
Models, reflection phases and amplitudes of the 2-bit coding unit cells. (**a**) The geometry of the first coding unit cell. (**b**) The geometry of the second coding unit cell. (**c**) The geometry of the third coding unit cell. (**d**) The geometry of the fourth coding unit cell. (**e**) The reflection phases of the 2-bit coding unit cells. (**f**) The reflection amplitudes of the 2-bit coding unit cells.

Owing to the maneuverability of the automatic optimization method, we design broadband 2-bit coding unit cells, which consist of four coding unit cells with phase difference of 90°. The procedure of the automatic-design is running with three steps, each invoking three *fitness* functions. Firstly, Eq. (4) is employed to provide a pair of unit cells with 180° out of reflection phases, as shown geometrically in Figs. 4(b) and (d). The corresponding reflection phases and amplitudes of the two unit cells are donated as blue and red lines in Figs. 4(e) and (f), respectively. Then based on one of the two unit cells, the second and third *fitness* functions are added to search for the unit cells whose reflection phases differ from the basic unit cells by 90° and 270° phases. Two *fitness* functions are depicted as

5 $$fitness={B}_{{90}^{\circ }\pm {10}^{\circ }}$$

and

6 $$fitness={B}_{{270}^{\circ }\pm {10}^{\circ }}$$

In the second and third steps, every particle just needs 256 numbers for one pattern because the basic unit cell is ready. A population size of 60 and the maximum iterations of 100 are predefined. Figures﻿ 4(a) and (c) represent the remaining two unit cells of the 2-bit coding units, and their reflection phases and amplitudes are respectively presented by the black and green lines in Figs. 4(e) and (f). We note a frequency band from 10.6 to 11.1 GHz, where the reflection phases of these unit cells meet the requirement of 2-bit coding elements with almost zero boil-off.

### Experiments and measured results

**Figure 5:**
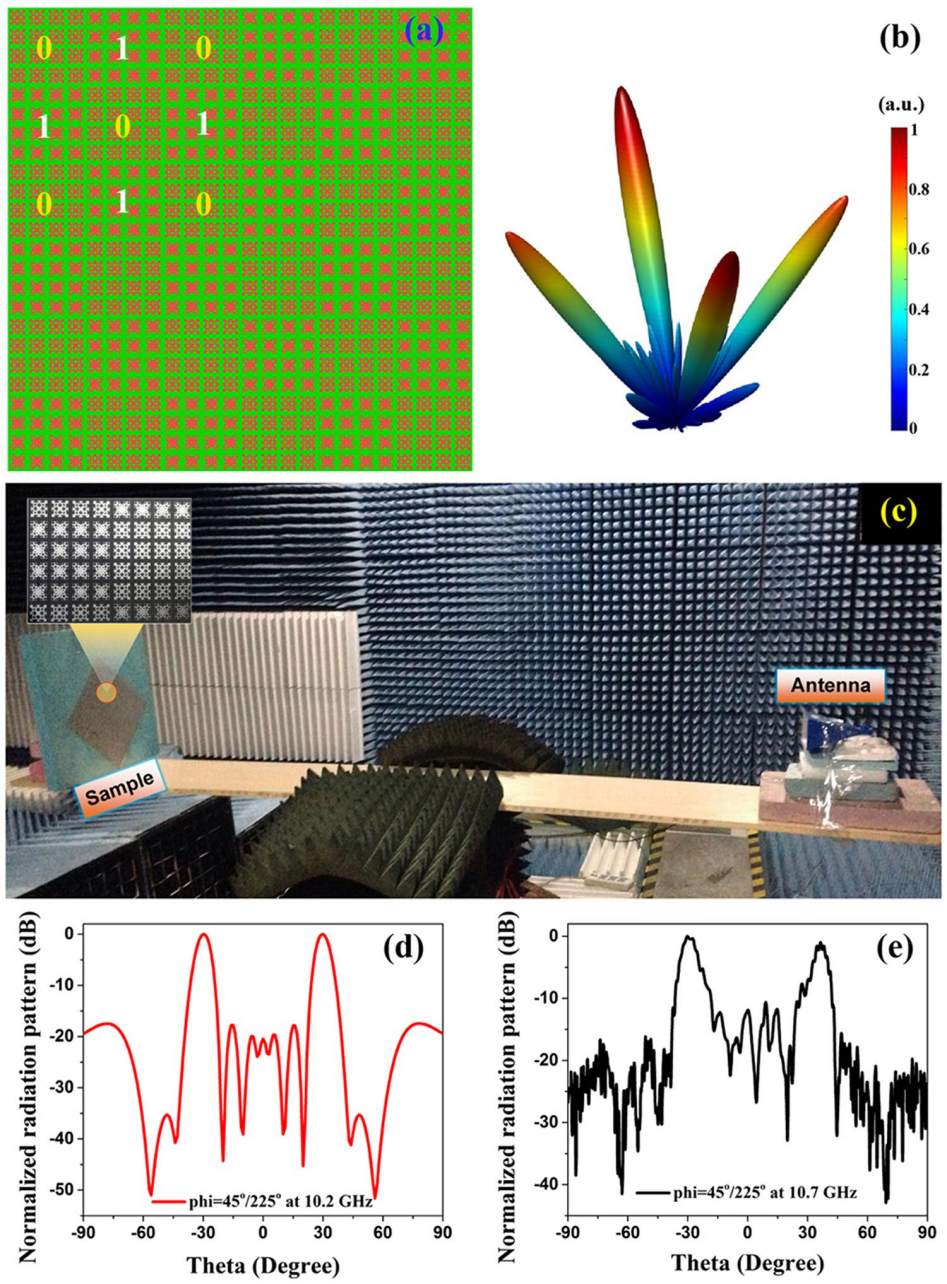
Simulated and measured scattering patterns for coding metasurface with 1-bit macro coding units. (**a**) Geometry of the coding metasurface with periodic coding sequence 0101…/1010…. (**b**) 3D full-wave simulation results of the coding metasurface. (**c**) The experimental setup for the far-field measurement in the microwave chamber. The (**d**) simulated, and (**e**) measured results of the 2D normalized radiation patterns at azimuth *phi* = 45°/225°.

Based on the 1-bit coding unit cells, we design and fabricate a coding metasurface with periodic coding sequence 0101…/1010… for checking the performance of the automatic design of the macro coding unit cells, in which 4 × 4 arrays of 1-bit coding elements (supper lattices) constitute the digital codes ‘0’ and ‘1’. The coding metasurface contains 6 × 6 supper lattices under the periodic arrangement of 0101…/1010…, as shown in Fig. 5(a). The three-dimensional full- wave simulation results of the coding metasurface are illustrated in Fig. 5(b) at 10.2 GHz in the working frequency band, and we can observe four main beams obviously and intuitively. The working frequency band of the simulated results ranges from 9.6 to 10.4 GHz, and the small frequency offset with the designed unit cells mentioned above is mainly caused by the coupling between different unit cells. Figure 5(c) depicts the physically testing environment in the microwave chamber where the receiving horn antenna is connected to a vector network analyzer (Agilent N5230C) for showing the far-field radiation patterns. Because the macro coding unit cells are designed with four-fold symmetry, the coding metasurface is insensitive to the polarization direction of the normally incident waves. In order to view the testing results clearly, the sample is placed on a square foam with the angle of 45°. From the two-dimensional normalized radiation patterns at azimuth *phi* = 45°/225° in Figs. 5(d) and (e), we apparently observe two main beams and lower side lobes at 10.2 GHz for simulated results and 10.7 GHz for measured results, respectively. We can also notice the discrepancy and frequency offset between measured and simulated results. The reason for the gaps may be caused by the machining tolerance in fabrication and the influence of the testing environment such as the misalignment of the center of horns and testing sample.

### 4-bit macro coding units

Since the random square lattice structures and their software-based automatic-approach have the characteristics of simplicity, convenience, and versatility, it is flexible to cope them with various complexities. There is a concise and fast procedure to create a series of multifarious reflection phases at specific frequencies. We choose 4-bit unit cells to cover the phase range of 360° at 10 GHz, so that the phase difference between two adjacent unit cells is assigned to 22.5° for uniformity. In order to facilitate the operation, the ideal reflection phases of 4-bit unit cells are programmed as

7 $$P(i)={210}^{\circ }-{22.5}^{\circ }\cdot i\,\,i=1,2\cdot \cdot \cdot 16$$

Based on the ideal values, the *fitness* function is set to

8 $$fitness=|P-P(i)|\,\,\,\,i=1,2\cdot \cdot \cdot 16$$

**Figure 6:**
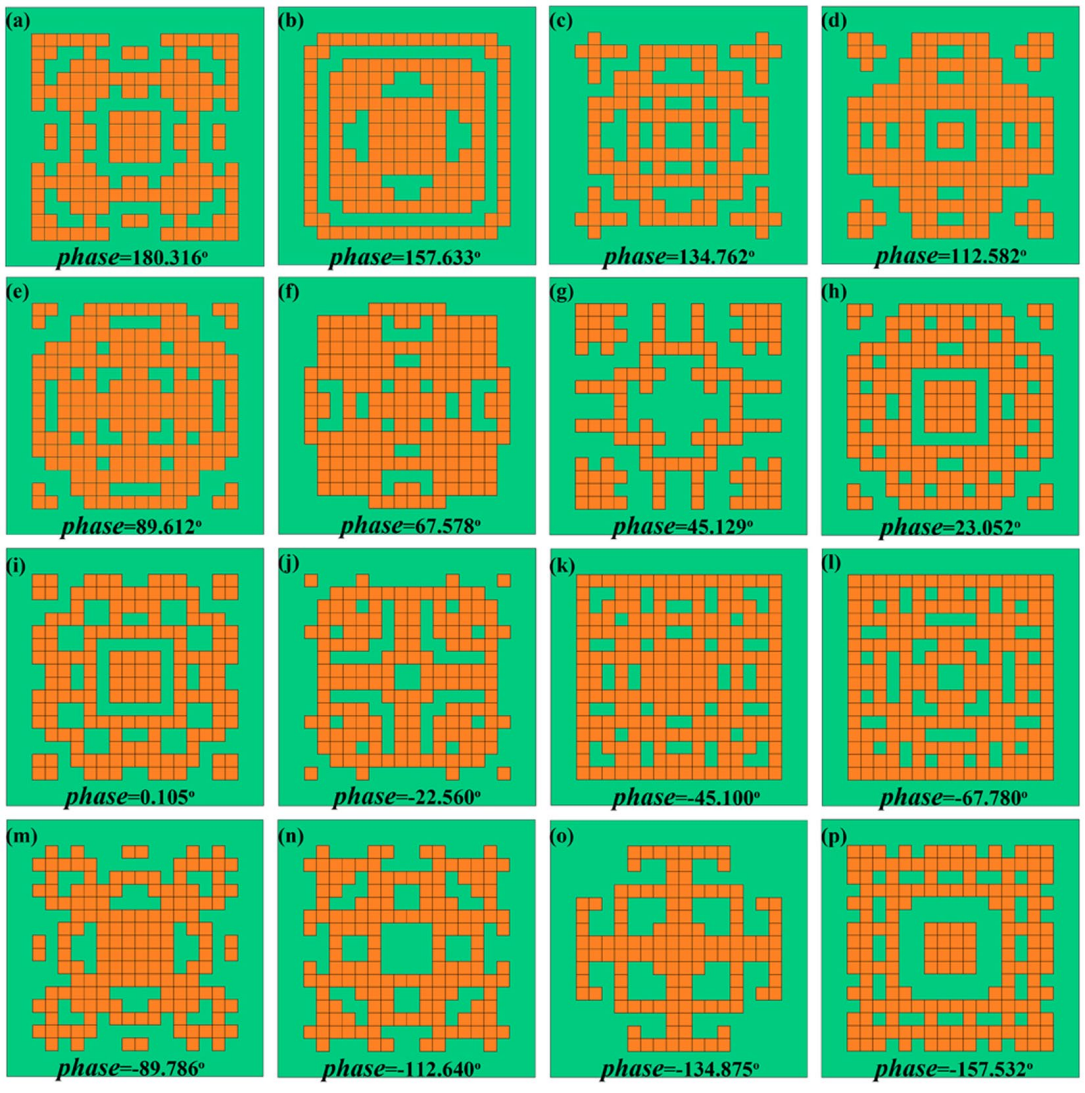
Coding patterns of the 4-bit macro coding unit cells. (**a**–**p**) Geometries of the 4-bit macro unit cells, in which the reflection phase of each unit cell is shown at the bottom of each panel.

Here, every particle is composed of 256 numbers for one pattern, and the initial population has 80 particles. After the maximum iterations scheduled for 100, the final optimized patterns of the 4-bit macro coding unit cells are described in Figs. 6(a–p), in which the inserted digits indicate the actual reflection phases, possessing 180.316°, 157.633°, 134.762°, 112.582°, 89.612°, 67.578°, 45.129°, 23.052°, 0.105°, −22.560°, −45.100°, −67.780^o^, −89.786^o^, −112.640^o^, −134.875^o^ and −157.532°. Compared with the ideal reflection phases, the maximum deviation of the automatic designed values is less than 0.6°.

**Figure 7:**
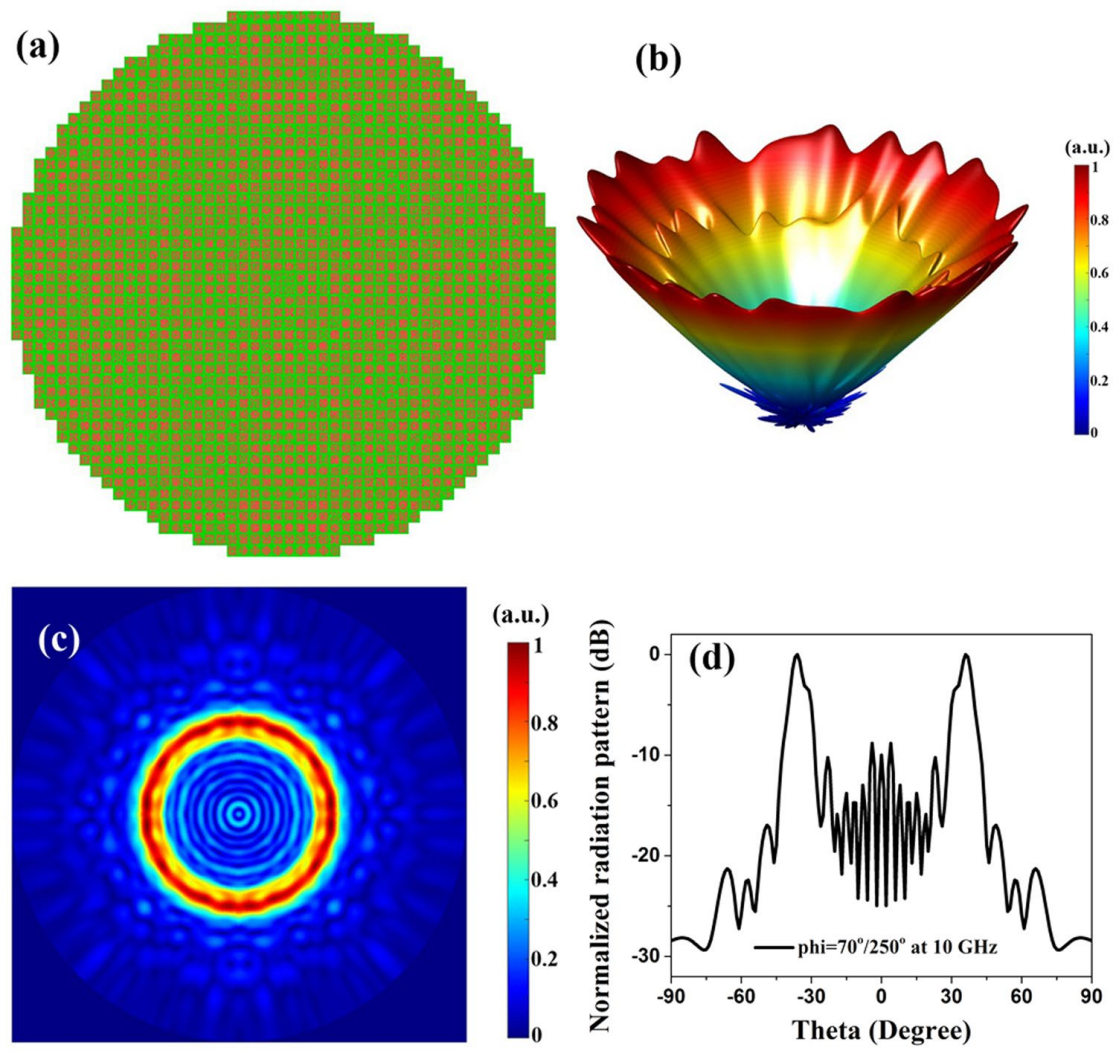
Simulated 3D and 2D scattering patterns for the quasi-circular metasurface with the circularly shaped radiation beam. (**a**) Geometry of quasi-circular metasurface. (**b**) The 3D full-wave simulation result of the metasurface at 10 GHz. (**c**) The simulated 2D scattering far-field pattern at 10 GHz. (**d**) The simulated 2D normalized radiation pattern in the Cartesian coordinate at 10 GHz.

**Figure 8:**
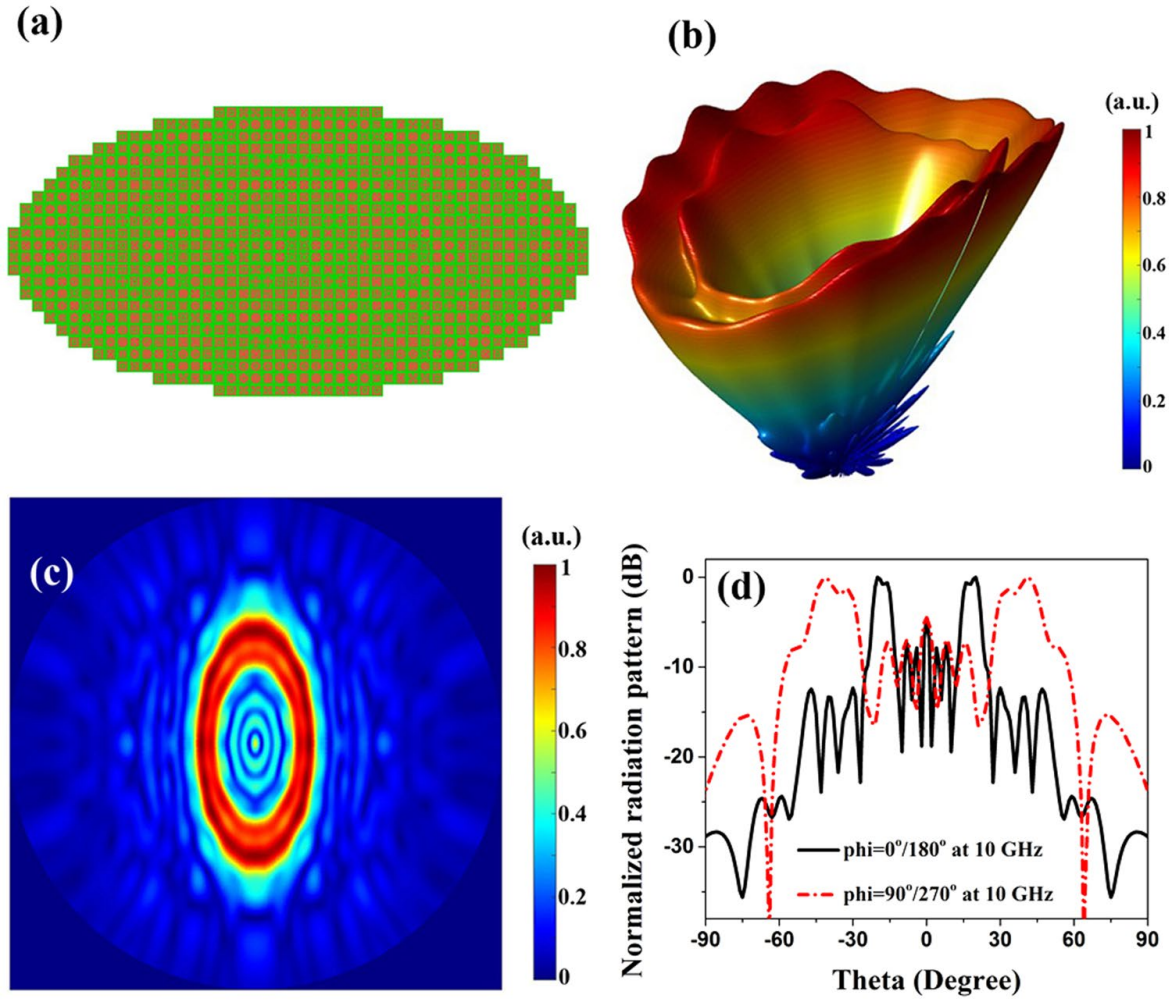
Simulated 3D and 2D scattering patterns for the quasi-elliptic metasurface with the elliptically shaped radiation beam. (**a**) Geometry of quasi-elliptic metasurface with the elliptically shaped radiation beam; (**b**) The 3D full-wave simulation result of the metasurface at 10 GHz; (**c**) The simulated 2D scattering far-field pattern at 10 GHz; (**d**) The simulated 2D normalized radiation pattern in the Cartesian coordinate at 10 GHz.

To verify the practicability of the automatic designs, we use such 4-bit macro coding units to fabricate two complicated functional metasurfaces, whose radiation beams are shaped as a circle and an ellipse, respectively. There are a variety of radiation effects by adjusting the arrangements of the macro unit cells. In order to radiate the corresponding shapes of beams, the required compensation phase for every unit cell is calculated as

9 $$\phi =-{k}_{\circ }\cdot \sqrt{f(x,y)}\cdot \,\cos (\theta )\pm 2n\pi \,\,\,n=1,2\cdot \cdot \cdot $$

where *k*_0_ is the free space wavenumber, *θ* is the pitch angle, *f*(*x*, *y*) is the function of the desired radiation beam (circle or ellipse in this design), and (*x*, *y*) is the coordinate of the macro unit. For the convenience of computing, the substrate center is selected as the origin of the coordinate system. Thus we just need to calculate the compensation phase of a quarter of the metasurfaces because of the symmetry of the circular and elliptical beams. Benefiting from the automatic and software design by Matlab calling CST MWS, it is easy to create various metasurface such as quasi-circular and quasi-elliptic metasurface we choose. As shown in Figs. 7(a) and 8(a), the radius of the quasi-circular metasurface is 240 mm, and two semi-axis of the quasi-elliptic metasurface are respectively 240 mm, 120 mm. For the sake of the circular and elliptical beams, the function *f*(*x*, *y*) is respectively chosen as

10 $$f(x,y)={x}^{2}+{y}^{2}$$

11 $${\rm{and}}\,\,\,f(x,y)={x}^{2}+\frac{{y}^{2}}{4}$$

Substituting Eqs. (10) and (11) into (9), respectively, we can calculate the compensation phase distributions of the circle metasurfaces with *θ* = 36° and ellipse metasurface with *θ* = 40° at 10 GHz. If the compensation phase is in the range between *P*(*i*)−11.25° and *P*(*i*) + 11.25°, the macro unit cell *i* should be placed at that position. Thus the arrangements of the two complicated functional metasurfaces are illustrated in Figs.  7(a) and 8(a), respectively, radiating the circularly and elliptically shaped beams. From the 3D full- wave simulation results in Figs. 7(b) and 8(b), it is intuitive to see a circular beam and an elliptical beam above the substrate at 10 GHz. Imperfect cone-shaped beams are mainly due to the coupling among macro unit cells and the discretization of the phase and location. Figures 7(c) and 8(c) depict the simulated 2D scattering far-field pattern, showing complete circle and ellipse beams. As shown the 2D normalized radiation pattern of quasi-circular metasurface in the Cartesian coordinate in Fig. 7(d) at azimuth *phi* = 70°/250°, we can observe that the radiation direction of main lobe is 36° in any azimuth angle. Similarly, from Fig. 8(d) for the 2D normalized radiation patterns of quasi-elliptic metasurface, the radiation direction of main lobe of major axis is 20° at azimuth *phi* = 0°/180°, and the radiation direction of main lobe of minor axis is 40° at azimuth *phi* = 90°/270°. Thus, both the quasi-circular metasurface with radiating the circularly shaped beam and the quasi-elliptic metasurface with radiating the elliptically shaped beam have excellent agreements to the designed values.

## Conclusion

Taking advantages of the novel optimization algorithm BPSO and the commercial software CST MWS, we can automatically obtain the satisfactory patterns of the random square lattice units (macro coding unit cells), which are composed of many square metal sub-blocks (micro coding unit cells) through different 2D arrangements. The software-based automatic-approach can achieve a maximum bandwidth for two macro coding units with phase difference of 180° and four macro coding units with phase difference of 90°, etc. In order to examine the performance of the software-based automatic-approach, we simulated and measured a coding metasurface with 0101…/1010… periodic coding sequence using the 1-bit macro coding elements. The numerical and experimental results demonstrated excellent performance of the 1-bit coding elements in a broad bandwidth. The software-based automatic-approach can also be used to search 4-bit macro coding units with sixteen fixed phases. We employ the 4-bit macro coding units to design two complicated functional metasurfaces, respectively, for radiating circularly and elliptically shaped beams. The full-wave simulated results are in excellent agreements with the design target. The proposed software-based automatic-approach for provides the versatility of coding units, and the novel optimization algorithm helps automatic designs of coding metasurfaces.

## Methods

A bridge is built up between the BPSO optimization algorithm and commercial software, CST MWS, for numerical simulation to achieve the optimal arrangement of the square metal sub-blocks. Figure 2 vividly describe the detailed process of the connection between Matlab (BPSO) and CST MWS. The BPSO optimization algorithm run in Matlab which generate initial population and update the speed and the location of particles in each step of iterations through judging the termination condition. Then, Matlab call CST MWS, and input the particle for updating the pattern and mode of unit cell in CST MWS. CST MWS simulates the phase and amplitude of each unit cell and output the information to evaluate the fitness. If the value of fitness cannot meet the termination condition, the program will update the particle for next iteration. Through multiple iterations and judging the termination condition, we can obtain the optimal pattern automatically of the desired target.

The 1-mm thin and flexible dielectric film (F4B) with the relative permittivity 2.65 (1 + 0.003i) is used to fabricate the metasurfaces. The metal layers with thickness 0.018 mm are adopted as a kind of annealed copper (electric conductivity *σ* = 5.8e + 007 S/m). As depicted in Fig. 5(c), the experiment is conducted in the microwave chamber. The sample and the transmitting antenna are placed on a rotary platform, which can automatically rotate of 360° in the horizontal plane. The receiving horn antenna,connected to a vector network analyzer (Agilent N5230C), is used to measure the radiated field to obtain far-field radiation patterns.
